# Complete genome sequences of paired isogenic *Burkholderia pseudomallei* isolated from a Thai pediatric patient with a urinary tract infection

**DOI:** 10.1128/mra.00470-24

**Published:** 2024-07-08

**Authors:** Sunisa Chirakul, Pintip Suchartlikitwong, Kritsakorn Saninjuk, Kiatichai Faksri, Kanitha Patarakul, Piroon Jenjaroenpun, Thidathip Wongsurawat

**Affiliations:** 1 Division of Bacteriology, Department of Microbiology, Faculty of Medicine, Chulalongkorn University, Bangkok, Thailand; 2 Center of Excellence in Antimicrobial Resistance and Stewardship, Faculty of Medicine, Chulalongkorn University, Bangkok, Thailand; 3 Department of Pediatrics, Center of Excellence for Pediatric Infectious Diseases and Vaccines, Faculty of Medicine, Chulalongkorn University, Bangkok, Thailand; 4 School of Science, Mae Fah Luang University, Chiang Rai, Thailand; 5 Microbial Products and Innovation Research Group, Mae Fah Luang University, Chiang Rai, Thailand; 6 Department of Microbiology, Faculty of Medicine, Khon Kaen University, Khon Kaen, Thailand; 7 Chula Vaccine Research Center (Chula VRC), Center of Excellence in Vaccine Research and Development, Chulalongkorn University, Bangkok, Thailand; 8 Siriraj Long-read Lab, Division of Medical Bioinformatics, Research Department, Faculty of Medicine Siriraj Hospital, Mahidol University, Bangkok, Thailand; Queens College Department of Biology, Queens, New York, USA

**Keywords:** *Burkholderia pseudomallei*, complete genome sequence, whole-genome sequence

## Abstract

*Burkholderia pseudomallei* is the causative agent of melioidosis, the disease endemic in Southeast Asia and northern Australia. We report complete genome sequences of paired isogenic *B. pseudomallei* isolated from a 12-year-old Thai male presenting with acute urinary tract infection before (SCBP001) and after (SCBP007) a decrease in susceptibility to ceftazidime.

## ANNOUNCEMENT

*Burkholderia pseudomallei* is the causative agent of melioidosis, primarily affecting the lung, spleen, and liver, while kidney infection is less common (1, 2). It is inherently resistant to many commonly used antibiotics; however, ceftazidime and meropenem are typically effective and recommended for treatment (2 – 4). Two isolates of *B. pseudomallei*, SCBP001 (collected prior to ceftazidime treatment) and SCBP007 (collected after the development of reduced susceptibility to ceftazidime), were isolated from urine samples of a Thai boy who was unaware of an underlying ureterovesical junction obstruction, using Cystine Lactose Electrolyte Deficient (CLED) and Blood agar. Single colonies were identified as *B. pseudomallei* using VITEK 2 XL colorimetric GN card (bioMérieux). Here, Oxford Nanopore Technologies (ONT) and Illumina sequencing were performed to study the whole genomes of these two isolates, with approval from the institutional review board (IRB) of the Faculty of Medicine, Chulalongkorn University. Patient consent was exempted (certificate of approval 0069/66).

The *B. pseudomallei* genomic DNA was extracted from both isolates grown in Lennox LB broth at 37°C with aeration using ZymoBIOMICS DNA kits (Zymo Research, USA). For ONT, the DNA library preparation was performed using the Rapid Barcoding kit (SQK-RBK004) prior to sequencing on a flow cell (version R9.4.1/FLO-MIN106) using the MinION MK1c (ONT). Raw signals from the sequencer were base called using a super high accuracy model (-c dna_r9.4.1_450bps_sup.cfg -r --trim_barcodes --barcode_kits SQK-RBK004) and demultiplexed using Guppy v.5.0.7 followed by adapter trimming using Porechop v.0.2.4 (5). ONT raw reads were filtered with >500 bases in length using NanoFilt v.2.8.0 (6). Quality control of reads was checked by NanoPlot v.1.28.1 (7), yielding 21× and 66× coverage for SCBP001 and SCBP007, respectively.

The same samples were prepared for short-read sequencing using the Illumina HiSeq platform (Omics Drive 99 Co., Limited, Singapore) according to the manufacturer’s protocol, generating 150 bp paired-end reads for each genome. Trimmomatic v.0.39 (8) was used to remove adaptors and poor-quality reads of Illumina reads which produced 195× and 164× coverage for SCBP001 and SCBP007, respectively.

Sequence data from both sequencing platforms were used for hybrid genome assembly using Unicycler v0.4.4 (9). The quality of the genomes was examined using QUAST v.5.0.2 (10). Taxonomic identification using GTDB-TK v.1.5.1 (11) identified it as *B. pseudomallei* based on average nucleotide differences and alignment fraction. Genome completeness estimation was performed through the identification of Benchmarking Universal Single-Copy Orthologs (BUSCO v.5.4.7) (12) using the Burkholderiales odb10 data set which contains 688 BUSCO markers. The genome sequence was annotated by the NCBI Prokaryotic Genome Annotation Pipeline v.6.6 (13). Default parameters were used except where otherwise noted.

The complete sequenced genome of *B. pseudomallei* strains consisted of two circular chromosomes (SCBP001; 3,964,321 bp and 3,199,400 bp and SCBP007; 3,964,337 bp and 3,198,821 bp) with an overall G + C content of 68.15%. Genome annotation results revealed 6,274 coding sequences, 76 tRNA genes, and 12 rRNA genes for SCBP001, and 6,273 coding sequences, 76 tRNA genes, and 12 rRNA genes for SCBP007 (Table 1). The completeness assessment of the assemblies showed that both strains have a 99.9% BUSCO score.

**TABLE 1:** Genome characteristics of two *B. pseudomallei* isolated from clinical specimens in Thailand

Sample	Genome accession number	Total sequence length (bp)	Number of sequences	N50 (bp)	GC content (%)	Number of CDSs^*a*^	Number of rRNAs	Number of tRNAs
SCBP001	JAQIYP000000000	3,964,321 and 3,199,400	2	3,964,321	68.15	6,274	12	76
SCBP007	JAQIYQ000000000	3,964,337 and 3,198,821	2	3,964,337	68.15	6,273	12	76

^*a*^ CDSs; coding DNA sequences.

## Data Availability

The complete genome sequence of *Burkholderia pseudomallei* SCBP001 and SCBP007 is available at the NCBI GenBank under accession numbers JAQIYP000000000 and JAQIYQ000000000 with BioProject number PRJNA922702  and BioSample accession numbers SAMN32672003 and SAMN32672004. Raw data reads are available at NCBI’s Sequence Read Archive under accession numbers SRR23095568 to SRR23095571.
